# Circadian rhythms in immunity and host‐parasite interactions

**DOI:** 10.1111/pim.12904

**Published:** 2022-02-16

**Authors:** Felicity K. Hunter, Thomas D. Butler, Julie E. Gibbs

**Affiliations:** ^1^ Centre for Biological Timing Faculty of Biology Medicine and Health University of Manchester Manchester UK

**Keywords:** circadian, innate immunity, parasite

## Abstract

The mammalian immune system adheres to a 24 h circadian schedule, exhibiting daily rhythmic patterns in homeostatic immune processes, such as immune cell trafficking, as well as the inflammatory response to infection. These diurnal rhythms are driven by endogenous molecular clocks within immune cells which are hierarchically coordinated by a light‐entrained central clock in the suprachiasmatic nucleus of the hypothalamus and responsive to local rhythmic cues including temperature, hormones and feeding time. Circadian control of immunity may enable animals to anticipate daily pathogenic threat from parasites and gate the magnitude of the immune response, potentially enhancing fitness. However, parasites also strive for optimum fitness and some may have co‐evolved to benefit from host circadian timing mechanisms, possibly via the parasites’ own intrinsic molecular clocks. In this review, we summarize the current knowledge surrounding the influence of the circadian clock on the mammalian immune system and the host‐parasitic interaction. We also discuss the potential for chronotherapeutic strategies in the treatment of parasitic diseases.

## 1 INTRODUCTION

Circadian rhythms refer to biological processes, observed in most organisms from cyanobacteria to humans, that recur naturally in an approximately 24 h cycle, driven by cell‐autonomous molecular oscillators referred to as ‘clocks’.^1^ The immune system is under circadian control, enabling organisms to anticipate daily threats from pathogens and gate the magnitude of the inflammatory response to pathogenic challenge. Rhythms in the host response to parasitic infection have been observed for millennia, including the periodicity of fever in malaria and the daytime somnolence of Human African Trypanosomiasis (HAT), commonly known as ‘sleeping sickness’. However, chronobiologists’ study of the host‐parasite interaction has only recently begun. Mounting evidence suggests that competing host and parasite rhythms lead to a complex interplay that impacts fitness and survival in both parties.^2^ In this review, we discuss circadian rhythms in immunity and explore the relative influence of host and parasite rhythms on the progression of parasitic disease. Given the huge pathological and socioeconomic burdens of parasitic disease, novel and effective clinical interventions are paramount. Here, we consider the role of chronotherapy as a future avenue for management of parasitic disease.

## 2 THE CIRCADIAN CLOCK

Circadian clocks are thought to have evolved across virtually all domains of life in response to 24 h environmental cycles generated by the earth's daily rotation on its axis. These molecular time‐keeping mechanisms enable organisms to anticipate and optimize their response to daily changes in light, temperature, nutrient availability and pathogenic threat, thereby increasing organismal fitness.^3^ Indeed, the fitness advantages of clocks have been demonstrated in a variety of organisms including simple prokaryotes^4^ and more complex multicellular plants.^5^ Furthermore, circadian misalignment in humans, as a result of jet lag or shift work, is known to increase the risk of various inflammatory diseases including asthma,^6^ COVID‐19^7^ and cancer.^8^, ^9^

**FIGURE 1:**
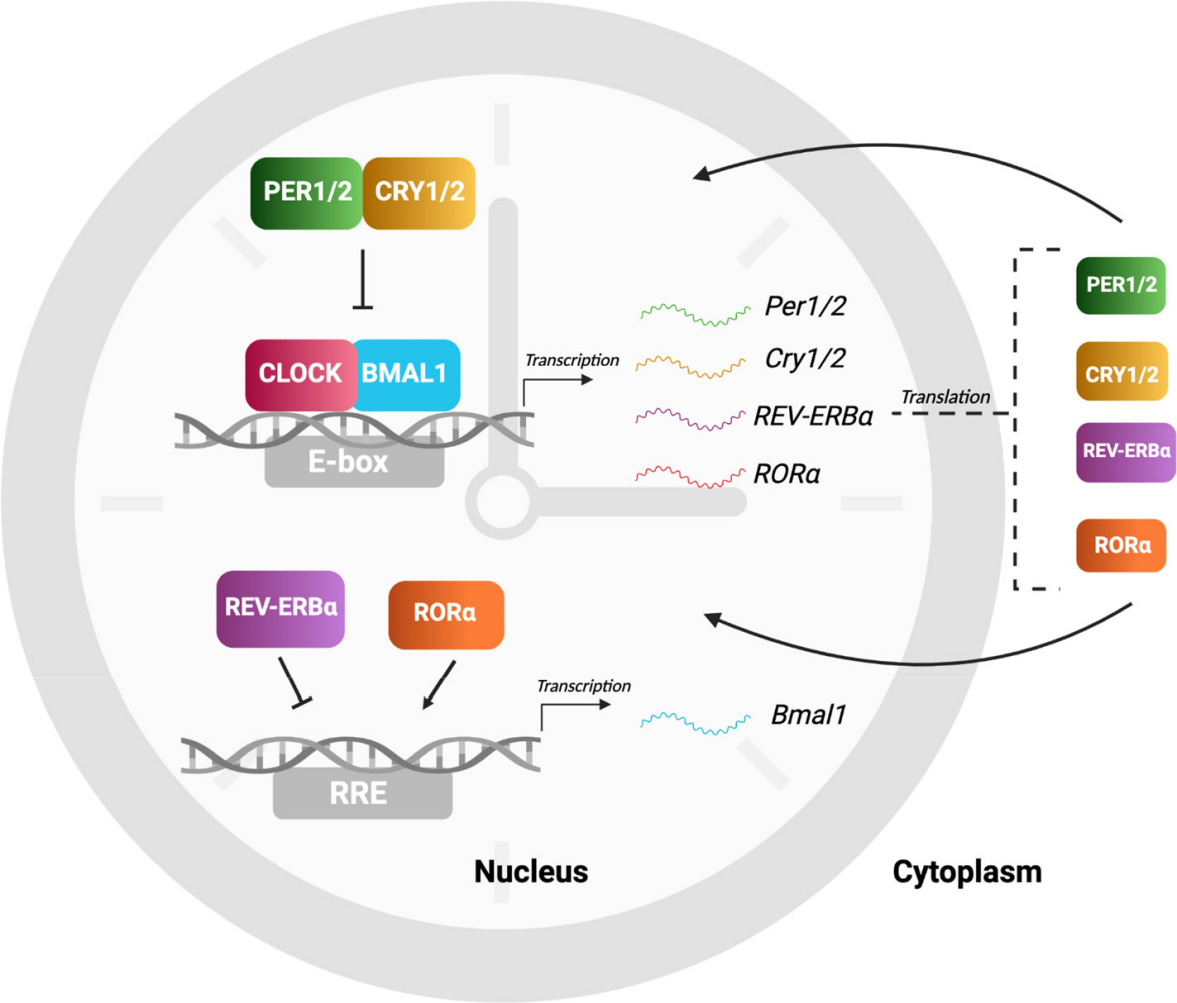
Molecular mechanisms of the mammalian circadian clock. The oscillatory mechanism of the mammalian clock relies on autoregulatory transcriptional‐translational feedback loops (TTFLs). The core TTFL involves the heterodimerization of the transcriptional activator proteins circadian locomotor output cycles kaput (CLOCK) and brain muscle arnt‐like 1 (BMAL1). The CLOCK‐BMAL1 heterodimer binds to the enhancer box (E‐box) region of the *Period (Per)* and *Cryptochrome (Cry)* target genes to activate transcription. PER and CRY proteins accumulate in the cytoplasm, translocate to the nucleus and negatively regulate the activity of the CLOCK‐BMAL1 heterodimer. An auxiliary TTFL involves the binding of the repressor protein *REV‐ERBα* and the activator protein retinoid‐related orphan receptor (ROR)α to the ROR‐response element (RRE) on the *Bmal1* promoter to regulate *Bmal1* expression and stabilize the core TTFL. Created in Biorender.com

Several well‐defined parameters are used to identify circadian clocks across taxa.^10^ Firstly, their capacity to oscillate with a period of approximately 24 h in the absence of external rhythmic cues, called zeitgebers (translated to ‘time‐givers’), in a phenomenon is called free‐running. Secondly, their ability to reset when misaligned via synchronization with external zeitgebers, the most studied of which is the light‐dark cycle, in a process is called entrainment. Thirdly, their capacity to maintain their 24 h period, despite fluctuations in the external temperature, in a process is called ‘temperature compensation’.^11^ Genetic comparisons have identified a common feature underpinning clock function across vastly diverse organisms is a network of ‘clock genes’ organized into autoregulatory transcriptional‐translational feedback loops (TTFL), involving a sequence of gene expression, accumulation and degradation with a duration of approximately 24 h.^1^ In mammals, the core TTFL comprises the proteins circadian locomotor output cycles kaput (CLOCK), brain muscle arnt‐like 1 (BMAL1), PERIOD1/2 (PER) and CRYPTOCHROME1/2 (CRY). An auxiliary TTFL involving the proteins REV‐ERBα and retinoid‐related orphan receptor (ROR)α stabilizes the core TTFL (Figure 1).

Whilst TTFLs are consistently observed across other phylogenetic kingdoms, the genes and proteins involved are often not conserved, suggesting that TTFLs may have emerged independently on multiple occasions throughout evolutionary time as products of convergent evolution.^12^ Contrastly, the cyanobacterial clock keeps time independently of transcription and translation.^13^, ^14^ Endogenous rhythms in the phosphorylation‐dephosphorylation of the clock protein KaiC remain robust in the presence of transcription and translation inhibitors^13^ and can be reconstituted *in vitro* with purified clock proteins together with ATP.^14^ These findings provided some of the earliest evidence of a non‐TTFL post‐translational oscillator sufficient for sustaining circadian rhythms. More recently, the discovery of non‐TTFL circadian oscillations in the oxidation state of peroxiredoxin (PRX) proteins was observed across all domains of life from humans to archaea, providing a possible universal marker for circadian rhythms.^15^, ^16^, ^17^ PRX proteins, antioxidants involved in removing toxic metabolic by‐products, are thought to have evolved ~2.5 billion years ago during the rapid rise of atmospheric oxygen, termed the Great Oxidation Event and may represent a common circadian ancestor, possibly reflected by the oxygen‐sensing PAS domains conserved in many eukaryotic clock genes.^18^ Intriguingly, PRX redox rhythms persist in the absence of a TTFL, both in human erythrocytes that lack a nucleus^16^ and organisms with mutations in TTFL components.^15^, ^17^ However, the phase and amplitude of PRX oscillations in TTFL mutants was perturbed and likewise organisms with mutations in PRX oscillator components exhibited disrupted TTFL function in plants and cyanobacteria.^15^, ^17^ Together, these findings suggest that TTFL and PRX timing systems may work in parallel and both are required for optimal circadian physiology. Recently, multi‐omic analyses have demonstrated a weak overlap between rhythmic gene transcripts and their encoded proteins, challenging the widely accepted view that timing instructions proceed linearly from transcription to translation.^19^, ^20^ Interestingly, core clock proteins have been shown to act post‐translationally to regulate protein synthesis and improve robustness of circadian outputs.^21^, ^22^ Whilst the search continues for a communicating factor between classic TTFLs and non‐TTFL mechanisms of circadian regulation, these data provide insight to the layers of complexity within circadian systems and encourages researchers to consider non‐TTFL mechanisms driving circadian observations, especially in cases where canonical clock gene homologues have yet to be identified, such as in most parasites.

Unlike unicellular organisms whereby a single clock drives rhythms in many different processes, multicellular animals possess self‐sustained clocks in almost every cell.^23^, ^24^ In mammals, there is a ‘master clock’ located in the suprachiasmatic nucleus (SCN) of the hypothalamus, comprising approximately 20,000 neurons, which synchronizes and coordinates the peripheral clocks in a hierarchical manner in line with the light‐dark cycle. Feeding time is also an influential timing cue, shown to be the dominant zeitgeber in some peripheral tissues.^25^ However, various other endogenous and extrinsic rhythmic cues can entrain peripheral clocks including body temperature^26^ and hormones.^27^ Peripheral clocks allow organisms to partition clock function amongst different cell types and coordinate tissue‐specific rhythms which can be integrated into the internal timing system to temporally regulate virtually all physiological processes including metabolism, body temperature and immunity.^28^

## 3 THE RHYTHMIC IMMUNE SYSTEM

The mammalian immune system is a complex network of sophisticated physiological mechanisms that evolved to protect the body against pathogens and promote wound healing. It can be divided into two main strategies: innate and adaptive immunity. The highly evolutionarily conserved innate immune response is the first line of defence, comprising both physical barriers, including the skin and mucosal surfaces of the respiratory and gastrointestinal tracts,^29^ and cellular components. The adaptive immune response comprises highly specialized systemic cells and mechanisms stimulated by pathogen exposure, either through infection or vaccination, to recognize specific ‘non‐self’ antigens, generate a pathogen‐specific response and form immunogenic memory to enable a rapid and efficient response upon re‐infection.^30^

The influence of the circadian clock in the regulation of the mammalian immune response was revealed more than 60 years ago in a landmark study which demonstrated 24 h variation in the host response to lethal infection.^31^ Subsequently, diurnal susceptibility to pneumococcal infection was shown to be altered in both blind and adrenalectomized mice, indicating the important contribution of light and adrenocortical hormones to rhythmic immune function.^32^ More recently, the requirement of the circadian clock for regulating various immune functions has been extensively characterized with the help of transgenic animal models harbouring cell‐specific clock gene deletions. For example, deletion of core clock gene *Bmal1* in murine macrophages disrupts protective rhythmic outputs, leading to a pro‐inflammatory phenotype with survival consequences.^33^ It is now known that processes including the synthesis and secretion of cytokines,^34^, ^35^ phagocytosis^36^ and immune cell trafficking^33^, ^37^, ^38^ are all under circadian control. Furthermore, strong time‐of‐day variation in human disease activity has been reported in several chronic inflammatory diseases including asthma^39^, ^40^ and rheumatoid arthritis.^41^

Rhythms in immunity are driven by intrinsic molecular clocks entrained to the 24 h day by endogenous and extrinsic rhythmic signals. Accordingly, functional clocks have been identified in most innate immune cells including natural killer cells,^42^ macrophages,^34^, ^43^ mast cells,^44^, ^45^ dendritic cells,^43^ eosinophils,^45^ monocytes,^33^ microglia,^46^ neutrophils^47^ and innate lymphoid cells.^48^, ^49^ Conversely, whilst clock machinery has been identified in some adaptive immune cells including CD4^+^ T cells^50^ and B cells^43^ the extent to which clock genes influence the diurnal rhythmicity of adaptive immune functions remains relatively elusive, with some conflicting findings reported.^50^, ^51^

There are a number of potential evolutionary benefits of a circadian‐regulated immune system. Firstly, it enables mammals to anticipate times of day when risk of pathogenic challenge is highest and mount proportional inflammatory responses when activated. Secondly, rhythmity allows immune components involved in a dynamic inflammatory response to coordinate signals that drive recruitment and activation.^52^ Thirdly, circadian rhythms coordinate the metabolic processes required for immune cell activation and prepare the immune system for exposure to rhythmic metabolic cues, such as feeding, that introduce fuel and non‐self antigens.^53^ A rhythmic immune system is vital for successful mammalian immunity. In the next section, we explore how circadian rhythms influence host‐parasite dynamics.

### 3.1 Rhythms in host immunity influence parasitic disease

Whilst a number of studies have demonstrated the circadian influence on pathogenic infections, the majority have focussed on bacterial infections with relatively few investigating the clock in the context of parasitic infections. Considering immune cells are targets for some intracellular parasites, an influence of rhythmic immunity on the progression of parasitic diseases is probable.^2^

#### 3.1.1 Rhythms in Leishmaniasis

**FIGURE 2:**
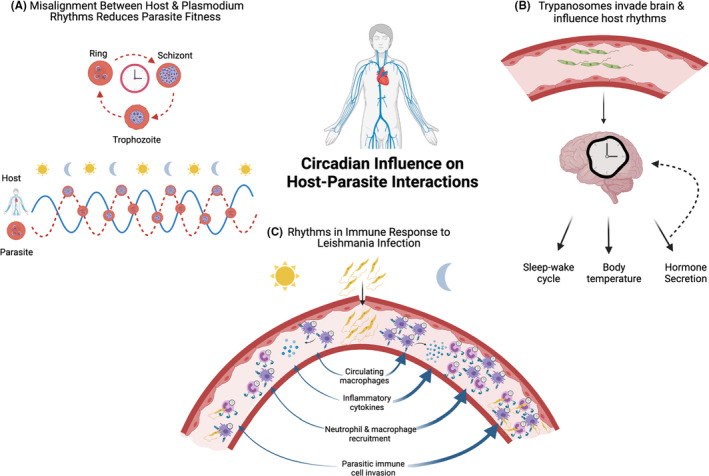
Circadian Influence on Host‐Parasite Interactions. (A) The intraerythrocytic developmental cycle (IDC) of malaria‐causing *Plasmodium* parasites is rhythmic, generally occurring in multiples of 24 h, depending on the *Plasmodium* species. Misalignment of the IDC schedule relative to host circadian rhythms imposes fitness consequences for parasites.^70^ (B) *Trypanosoma brucei* parasites, which cause Human African Trypanosomiasis, invade brain regions involved in circadian regulation such as the hypothalamus, disrupting host circadian‐regulated processes including sleep, body temperature and hormone secretion.^94^, ^100^ (C) Time of day of infection with *Leishmaniasis*‐causing *Leishmania* parasites affects the magnitude of the inflammatory response, due to the circadian regulation of host immunity.^55^, ^56^ Rodents infected at night exhibit greater parasitic burden than those infected during the day due to a higher number of circulating macrophages exposed to parasites at night, leading to increased levels of cytokines and greater recruitment of neutrophils to the site of infection, which are invaded by parasites. Created in Biorender.com

Leishmaniasis, caused by protozoan *Leishmania*, has been a focus for chronobiologists due to the nocturnal biting activity of its vector, the sandfly,^54^ as well as the *Leishmania* parasite's intracellular amastigote stage targeting circadian‐regulated neutrophils and macrophages.^3^ Time of day of subcutaneous footpad injections with *Leishmania* parasites influenced the magnitude of the inflammatory response, including footpad swelling and parasite load, both in hamsters maintained under light:dark conditions^55^, ^56^ and in mice under constant darkness.^55^, ^56^ In mice, increased parasitic burden during infection at night was associated with elevated neutrophil and macrophage recruitment to the site of infection in the skin compared to daytime infections.^56^ Given neutrophils are the hosts of early‐stage amastigotes,^57^ rhythmic neutrophil infiltration, driven by rhythmic expression of chemokines by macrophages, was postulated to cause the observed rhythms in parasite load^56^ (Figure 2). Supporting this, targeted *Bmal1* deletion in neutrophils and macrophages was sufficient to abolish the endogenous rhythms observed in immune cell recruitment and parasite burden.^56^ Together, these findings highlight the important role of the molecular clock within circulating immune cells in gating the magnitude of the inflammatory response to infection by *Leishmania* parasites.

#### 3.1.2 Rhythms in helminth infection

Researchers have demonstrated that the time of day of infection with murine infective nematode helminth *Trichuris muris* (*T*. *muris*) influences both the type of immune response generated and the timing of subsequent parasitic worm expulsion 21 days later.^58^ Mice, infected with *T*. *muris* in the early morning at Zeitgeber Time 0 (ZT0), exhibited the typical helminth‐induced Th2‐polarized immune response, determined by their cytokine and antibody profiles. Conversely, mice infected at ZT12 exhibited a non‐classical Th1‐polarized immune response and less efficient worm expulsion. Interestingly, this time‐of‐day influence on worm expulsion was abolished in mice with *Bmal1*‐deficient dendritic cells (DCs). Furthermore, RNAseq analysis of *Bmal1*‐deficient DCs challenged with the parasitic antigen *in vitro* demonstrated that the Th1‐associated cytokine profile and signalling pathways are BMAL1‐dependent. This study revealed the influence of the DC clock in temporally regulating the Th2/Th1 balance and the kinetics of worm expulsion in *T*. *muris* infections. Taken together, these findings may represent a fitness advantage of a rhythmic immune system for regulating the magnitude and type of immune response in an attempt to align immune activation with anticipated peaks and troughs in pathogen exposure.

#### 3.1.3 Rhythms in malaria

Periodic episodes of fever represent a classic clinical sign in malaria patients, generally occurring every 24, 48 and 72 h, depending on the infecting *Plasmodium* species.^59^ These recurrent paroxysms are a consequence of the successive, synchronous progression of blood‐stage *Plasmodium* parasites through the intraerythrocytic developmental cycle (IDC), ending in the simultaneous bursting of erythrocytic schizonts and release of transmissible merozoites.^60^ Despite the variability of the IDC duration between *Plasmodium* species, the fact that it is commonly a multiple of 24 h suggests it is circadian in nature. However, the relative host and parasite control of this synchrony, as well as the adaptive advantages for both parties, has been the subject of debate, with several hypotheses proposed over the years.^59^, ^60^, ^61^, ^62^, ^63^

Early studies centred around the assumption that *Plasmodium* parasites lack intrinsic timing mechanisms and instead rely on host circadian output, particularly rhythms in the host innate immune response, to synchronize parasites and organize the timing of the IDC.^59^, ^64^ Supporting this idea, parasites grown in culture lose synchrony in the absence of extrinsic host signals.^65^ Additionally, shifting normal host rhythms alters the timing of the IDC.^66^ Furthermore, early‐stage parasites responsible for eliciting the innate immune response are less vulnerable to its inflammatory insult compared to later‐stage parasites undergoing cell‐division, suggesting that the host immune response may gate the survival of parasites at different stages, thus generating rhythms in the cycle.^60^, ^64^, ^67^ However, recent findings challenge this hypothesis by revealing that timing of innate febrile response aligns with erythrocytic schizogony, regardless of the time of day it occurs.^68^ Furthermore, the same study showed delaying schizogony delays host fever response to the same extent.^68^ These findings instead suggest that the parasite is responsible for driving the timing of the innate febrile response, rather than the host clockwork. Therefore, whilst rhythms in the inflammatory response may reinforce existing rhythms in the IDC, they are unlikely to drive this phenomenon.^69^ Indeed, it seems that parasites are not simply bystanders of the rhythmicity observed during mammalian host infection.

## 4 HOW PARASITES TELL TIME

Complexity in the circadian regulation of the host‐parasite interaction arises from the possibility that, rather than solely providing constraints to parasite survival, host rhythms may also provide opportunities for parasites with rhythmic capabilities to increase their fitness. For example, by aligning with host rhythms, parasites may exploit temporally regulated host resources such as nutrients to enhance survival or host cells to enhance infectivity. Additionally, temporal coordination may facilitate the parasites’ evasion of rhythmic immune mediators to reduce the likelihood of pathogen clearance. Supporting this, induced mismatch in the IDC relative to host circadian timing has been shown to have costly consequences for *Plasmodium* parasites in‐host survival and between‐host transmission^70^ (Figure 2). However, several long‐standing questions regarding exactly how and why parasites tell time have intrigued evolutionary ecologists.^69^, ^71^, ^72^ Firstly, to what extent are host rhythms important for rhythms in parasites and which outputs are contributing? Secondly, how might parasites benefit from aligning their rhythms to the host? Thirdly, do parasites have their own intrinsic timing mechanism, and if so, what is it composed of?

Since clocks have been detected in virtually all living organisms, it is important to investigate potential intrinsic timing mechanisms in parasites. Whilst observations that *Plasmodium* parasites de‐synchronize when cultured *in vitro* cast some doubt on the presence of an intrinsic clock,^73^ a similar phenomenon is observed in single‐cell cultures of clock‐harbouring mammalian cells which rapidly de‐synchronize in the absence of zeitgebers such as the light‐dark cycle and temperature.^74^, ^75^ It has been previously suggested that, rather than generating their own self‐sustaining oscillations, parasites might tell the time using a more basic system, such as a ‘plastic strategy’^76^ whereby they simply respond to rhythmic output in their environment, such as an immune factor, a hormone or metabolite, which may allow for more efficient survival, with lower energy demands.^69^, ^71^ Notably, many parasites experience complex multi‐host lifestyles, often subject to highly dynamic daily extrinsic cycles throughout their life cycle, both in the external abiotic environment and within biotic vectors and hosts. Indeed, there is evidence that some parasites are sensitive to the timing of daily rhythms in both their abiotic environment and in their vectors, which may allow them to enhance survival and transmission respectively.^71^, ^72^ For example, 24 h periodicity in the number of filarial nematode (roundworm) parasites in the rodent host bloodstream corresponds to vector biting habits which may serve to enhance transmission.^77^ Similarly, infective *Schistosoma mansoni* (*S*. *manson*i) platyhelminth (flatworm) larvae (cercariae) are emitted from their intermediate mollusc hosts at a strain‐specific time of day, correlated to the behaviour of their definitive mammalian hosts, with human infective strains emerging during the day and rodent infective strains emerging at night. This phenomenon may have evolved to optimize host encounters and increase transmission.^78^, ^79^ Recent transcriptomic analysis has revealed rhythmic gene expression in adult *S*. *mansoni* parasites residing within the mesenteric vasculature of rodent hosts, an environment exposed to daily changes in temperature,^80^ pressure,^81^ oxygen levels,^82^ glucose levels^83^ and leukocytes^2^ which may all contribute rhythmic signals to influence parasite function.^84^ Indeed, approximately 2% of *S*. *mansoni* genes exhibited time‐of‐day dependent expression, including genes involved in stress response peaking during the night and genes involved in metabolic activity peaking during the day, correlating with the host's active and resting phase respectively. This temporal partitioning of physiological processes may help the parasite cope with time‐of‐day environmental challenges including host immunity, enhancing parasitic survival. However, genomic analysis failed to identify homologs of generally conserved core clock genes.^84^ It is likely that parasites either use novel TTFLs with as yet undiscovered clock genes or non‐TTFL mechanisms, evolved to keep time and anticipate, rather than react to, changes in their environment. In the following sections, we discuss the recent evidence for intrinsic clocks in specific parasites.

### 4.1 Clocks in *Trypanosoma brucei*

The first evidence for an intrinsic parasitic clock was demonstrated in the kinetoplastid protozoan parasite *Trypanosoma brucei* (*T*.* brucei*), the causative agent of Human African Trypanosomiasis (HAT).^85^ 4‐hourly RNA sequencing of *T*. *brucei* cultured in free‐running conditions (in the absence of extrinsic rhythmic cues) demonstrated oscillations in approximately 10% of the *T*. *brucei* transcriptome across two life‐cycle stages. Furthermore, oscillating transcripts were also detected from serial sampling of blood parasites in a mouse model of *T*. *brucei* infection. Notably, oscillating genes mapped to metabolic pathways, which correlated with rhythmic intracellular ATP levels, highlighting a potential adaptive advantage of intrinsic rhythmicity for parasite energy efficiency. Together, these findings suggest that *T*. *brucei* controls its daily schedule via an intrinsic timing mechanism. Of interest, *in vitro* parasites were entrainable to temperature but not light, which suggests *T*. *brucei* parasites may use the host's body temperature as a zeitgeber to coordinate its metabolism with host activity. As the genetic components of this clockwork remain elusive, the authors suggest that the *T*. *brucei* clock may take the form of a post‐transcriptional oscillator, due to the inclusion of both cycling and non‐cycling genes within co‐transcribed polycistronic units.^85^

### 4.2 Clocks in malaria parasites

More recently, evidence of a putative intrinsic oscillator in *Plasmodium* parasites has emerged.^86^, ^87^, ^88^ 24 h rhythms in *Plasmodium chabaudi* (*P*. *chabaudi*) gene expression and IDC rhythms persisted in arrhythmic murine hosts with global genetic deletion of core clock genes *Cry1*/*Cry2*, indicating independence from host rhythms.^88^ However, unlike parasites in wild‐type mice which remained robust and synchronous, a reduction in synchrony was observed in parasites in arrhythmic mice, which was eventually lost after the peak of parasitemia (8–9 days post‐infection). Similar to what is known for both mammalian fibroblasts^73^ and *T*. *bruce*i parasites,^85^ these findings indicate that *Plasmodium* parasites also require external timing cues to entrain their rhythms and maintain population synchrony. Supporting this, another study reported 57% of *P*. *chabaudi* genes exhibit 24 h rhythms in transcription, including those involved in important processes such as DNA replication.^85^ Notably, this rhythmicity was lost in the majority of these genes when parasites were mismatched from the host.^86^ However, since *P*. *chabaudi*'s IDC lasts 24 h, it is difficult to distinguish IDC genes with putative ‘clock genes’. To overcome this, researchers cultured *P*. *falciparum*, whose IDC lasts 48 h, in free‐running conditions.^86^, ^87^ Here, genome‐wide screening revealed that 6% of *P*. *falciparum* genes exhibit robust self‐sustaining 24 h rhythms in expression.^86^ Of these genes, many mapped to the same processes that exhibited reduced rhythmicity in mismatched *P*. *chabaudi* infections, suggesting the presence of an intrinsic timing mechanism sensitive to host circadian output. Of interest, transcriptomic analysis of four different *P*. *falciparum* strains revealed a broad range of *in vitro* periods ranging from 36 to 54 h; however, within‐strain cycle length variation and rate of asynchrony in culture was comparable to that of known circadian systems.^86^ Furthermore, *P*. *chabaudi* parasites can adjust their IDC length to align to changes in host rhythms. For instance, when ‘long period’ mutant mice are infected under constant darkness, parasites gradually delay the IDC rhythm to match the long period of 25.7 h.^88^ Similarly, parasites shorten the IDC length by 2–3 h during infection of mismatched hosts and realign within 5–6 days.^89^ Together, these findings suggest that *Plasmodium* parasites prioritize functional plasticity over robust rhythmicity to enhance alignment to host rhythms.^88^ Overall, these findings suggest that *Plasmodium* parasites can control the timing of their IDC to some degree to align with host rhythms.

Recent studies have demonstrated alignment of the *Plasmodium* IDC to rhythms in host feeding and metabolism rather than the circadian output generated by the host clock.^68^, ^90^, ^91^ These observations stimulated the search for a rhythmic circulating product of host digestion such as a metabolite or hormone that provides a timing cue for the temporal organization of the IDC. Indeed, a recent large‐scale metabolic screen of promising candidates, which cannot be synthesized by the parasite and whose rhythms are coordinated both with host feeding and the IDC, identified the amino acid isoleucine as being sufficient for scheduling the IDC.^69^ Removal and re‐addition of isoleucine caused the IDC to pause and restart at the same rate, respectively, with no costs to parasite survival. These findings suggest that parasites may use isoleucine as a daily timing cue to align the IDC with host rhythms in order to maximize nutritional gain. Notably, disruption of serpentine receptor 10 (SR10), a transmembrane G‐protein coupled receptor which exhibits 24 h rhythms in *Plasmodium species*, has been shown to shorten the duration of the IDC.^86^ Therefore, whilst the mechanism by which parasites sense isoleucine and integrate this signal with their timing system to influence the IDC schedule remains unknown, the authors suggested that SR10 might be involved in this process. Probing the sensitivity of SR10 to isoleucine and the effect on downstream signalling pathways could be key to unravelling the molecular mechanisms of the *Plasmodium* clock.^69^

In summary, mounting evidence suggests that parasites possess intrinsic time‐keeping mechanisms, entrained by host and environmental cues, to generate endogenous rhythms which align with host circadian timing mechanisms and likely enhance infectivity and survival. However, further work is required to elucidate the molecular components involved in generating these intrinsic rhythms and their relative contribution to the temporal variation observed in parasitic disease.

## 5 PARASITES INFLUENCE HOST CIRCADIAN RHYTHMS

Evidence of intrinsic clocks in parasites elevates them above bystanders in the host‐parasite interaction, and there is evidence that some parasite infections can influence circadian host physiology, for example in *T*. *brucei*‐induced Human African trypanosomiasis (HAT), also known as ‘sleeping sickness’ due to its characteristic disruption of the sleep‐wake cycle (Figure 2). In stage one of the *T*. *brucei* infection, parasites populate the bloodstream, lymphatic system and interstitial space of several organs.^92^, ^93^ Stage two begins when parasites emerge in the cerebrospinal fluid, signifying central nervous system invasion, followed by parasite accumulation and inflammatory cell infiltration to brain regions involved in circadian regulation, including the hypothalamus.^94^ Accordingly, as well as the characteristic disruption to the timing and architecture of sleep, impairment of other circadian‐regulated processes including body temperature^95^ and hormone secretion^96^, ^97^ has been reported in HAT.

Observations dating back to the 1800s revealed fragmented sleep in HAT patients, characterized by short spells uniformly distributed across 24 h, with total sleep duration not dissimilar from healthy people.^98^ Interestingly, this predated the discovery of the circadian clock, with HAT not recognized as a circadian disorder until EEG monitoring of the sleep‐wake cycle became possible in developing countries over a century later.^99^ Since then, animal models have enabled researchers to extensively probe the influence of *T*. *brucei* infection on circadian mechanisms. As reported in HAT, rhythms in activity, sleep and body temperature are disrupted in *T*. *brucei*‐infected rodents, validating their use as models.^100^, ^101^ In free‐running conditions, infected mice exhibit period shortening in activity, phase advance in activity onset and reduced ability to re‐entrain to light:dark conditions, indicating clock disruption.

Given that inflammation interferes with both sleep and the clock,^102^, ^103^ it is possible that the observed circadian disruption associated with *T*. *brucei* infection could be attributed to the induced Th1‐skewed adaptive immune response. Supporting this, stimulation of rat SCN brain explants with interferon‐γ and LPS disrupts diurnal variation in electrical activity to a similar extent as *T*. *brucei* infection.^104^ Alternatively, there is evidence to suggest that *T*. *brucei* infection influences circadian systems via peripheral endocrine signals that feedback to the SCN, independently of the immune system. For instance, 42 days post‐infection in rats, *Per1* period was shortened by 30 min in the pituitary gland and *Clock* and *Bmal1* expression levels were reduced in pineal gland and spleen explants respectively.^101^ Notably, rhythmic *Per1* expression was maintained in 79% of SCN explants. This observed robustness of the central clock likely reflects the extent of parasite invasion at this infection stage. Unlike peripheral tissues, which are rapidly infiltrated by parasites, the SCN is shielded by the blood‐brain barrier delaying parasite invasion.^105^ Supporting this, period shortening in activity levels was detected within 10 days of infection in mice, when few parasites have infiltrated the brain, with progressive shortening observed in later stages.^100^ Furthermore, as early as day 6, phase advance of PER2 protein expression was observed in adipose tissue, with a two‐hour shortened PER2 period observed at day 20. Significantly, these effects were abolished when mice were treated with suramin at day 60 to eliminate parasites in the periphery, with PER2 periods recovering to reflect those in control mice. Intriguingly, unlike the previously mentioned findings of robust SCN *Per1* oscillations in rats at day 42 by Lundkvist and colleagues, SCN explants harvested at day 60 in sumarin‐treated mice exhibited 30 min period shortening of PER2, corresponding to the stage when disruption to activity and body temperature period was reported. As sumarin cannot eliminate parasites in the brain, due to inability to cross the blood‐brain barrier, this indicates that there is sufficient parasite burden in the rat brain at day 60 for central clock disruption. In summary, these findings suggest that *T*. *brucei* parasites can influence host clocks peripherally and centrally, the extent of which is driven by the duration and burden of infection.

To date, there is little evidence to support other parasites influencing the circadian period of mammalian hosts. Despite inducing a similar immune response as *T*. *brucei*, mice infected with *P*. *chabaudi* had normal circadian rhythms, suggesting period shortening is not only independent of the immune system but may also be *T. brucei*‐specific.^100^ However, a recent study of three different *P*. *chabaudi* genotypes during four different segments of disease (asymptomatic, moderate, severe, recovery) reported short‐term rhythm disturbance in activity and body temperature in a parasite‐genotype dependent manner.^106^ This finding highlights the possibility that host circadian disruption is a genetically variable parasite trait which may be selected for and suggests that circadian disruption could be overlooked when considering disease impact as a whole rather than segmentally. Interestingly, the fungal parasite *Ophiocordyceps unilateralis s*.*l*. is known to manipulate the diurnal behaviour of carpenter ant hosts, driving the ants to leave their nest at a different time of the day and reach a more elevated position when they die, improving parasite spore transmission.^107^, ^108^ However, the fitness advantage driving the evolutionary selection of this trait in protozoan parasites is not entirely clear. There are obvious benefits for a parasite that is able to deregulate host immunity or metabolism to evade initial immune response and exploit nutritional resources; however, this must be balanced against previously described evidence demonstrating that perturbations to host rhythms has fitness costs for *Plasmodium* parasites.^70^ Future work is required to understand whether and how protozoan parasites benefit from influencing host rhythms.

## 6 CHRONOTHERAPY OF PARASITIC INFECTIONS

Chronotherapy, also known as circadian therapy, focusses on manipulation of the molecular clock or its rhythmic outputs to improve human health. In non‐communicable diseases including hypertension, cancer and asthma, there is growing interest in time of drug administration as a method to maximize efficacy and minimize side effects; however, evidence is lacking in parasitic diseases.^109^ Morbidity and mortality from parasites are still major worldwide challenges, and chronotherapy may have the potential to improve outcomes in a cost‐effective way by modifying administration schedules of current anti‐parasite drugs. Whilst it would be inappropriate for physicians to delay treatment of patients presenting with acute, sometimes life‐threatening infection, current drug therapy could be optimized in stabilized patients and milder cases, with the aim of accelerating recovery and reducing risk of complications.

Parasitic chronotherapy research has focussed on malaria, which is known to have stage‐dependent sensitivity to drug treatments, such as chloroquine, which disrupts lysosomal activity and autophagy. Mice infected with *P*. *chabaudi* had higher blood chloroquine levels when administered during the resting phase, compared to the active phase.^110^ Here, the resting phase correlated with parasites predominantly at mid‐trophozoite stage; however, there were no phenotypic outcomes in this pharmacokinetic study and no work investigating the mechanisms driving variation in blood chloroquine levels. In Madagascan patients presenting with *P*. *falciparum* malaria, the proportion of trophozoites detected on blood films prior to chloroquine treatment correlated with efficacy of trophozoite clearance, as calculated by the number of trophozoites detected on blood film the following day, after chloroquine treatment.^111^ However, time of day of sampling was not recorded in this non‐blinded, non‐randomized trial. It should be noted that *P*. *falciparum* is now resistant to chloroquine in many parts of the world and therefore chloroquine is no longer indicated as a treatment.^112^

Artemisinin combination therapy is currently the best available treatment for malaria.^112^ In mice infected with *P*. *chabaudi*, artemisinin showed greater efficacy in reducing parasite numbers when administered at the trophozoite stage, compared to the ring stage. This observation may be due to the rings’ ability to tolerate exposure to haem‐activated artemisinin, suggesting the temporal dependence of drug effectiveness is due to time‐of‐day sensitivity of parasites.^113^ This effect was enhanced when parasite rhythms were misaligned with host rhythms, with rings becoming less drug‐sensitive and trophozoites becoming more drug‐sensitive, further highlighting the parasitic fitness benefits of host alignment.^70^ Treatment of *in vitro* *T*. *brucei* cultures with anti‐protazoal suramin also leads to a time‐of‐day susceptibility.^85^ If these results are translatable to human infective strains, timing of artemisinin to target trophozoites might be more beneficial than timing administration to host phase. As parasitic drug resistance continues to be a major issue in malaria treatment, anti‐malarial chronotherapy and methods to uncouple host‐parasite rhythms provide exciting avenues to potentially improve outcomes for patients with malaria.

Notably, the World Health Organization has recently recommended rollout of the first malaria vaccine, RTS,S, for children in sub‐Saharan Africa, via a schedule of 4 doses from 5 months old.^114^ Evidence for vaccine chronotherapy is sparse, but a randomized controlled trial of influenza vaccination in the UK revealed a greater antibody response in those vaccinated in the morning compared to afternoon,^115^ which may be related to circadian‐regulated toll‐like receptor 5 expression.^116^, ^117^ Whilst there are no data for vaccine chronotherapy related to patient morbidity or mortality, the possibility of optimizing time‐of‐day administration to boost immune response should be considered in trials evaluating malaria vaccines. Chronotherapy is still in its infancy, but methods to optimize drug sensitivity must be investigated and time of day should be considered in therapeutic clinical trials.

## 7 CONCLUDING REMARKS

Organisms temporally arrange daily processes via circadian rhythms to maximize energy efficiency, survival and replicative potential. Convincing evidence of intrinsic clocks in mammalian parasites has recently emerged, particularly within protozoan parasites such as *T*. *brucei* and *Plasmodium*. This will likely lead to discovery of the specific components driving these timing mechanisms, as well as further examples of parasitic clocks. When hosts and parasites meet, host homeostatic rhythmic processes are disrupted with consequences for immune response and host health. Parasites are not bystanders in this interaction, but further work is required to understand how and why parasites interact with host circadian rhythms to improve their fitness. As research in this area expands, focus can shift to the translational potential of parasite chronotherapy with the ultimate aim of improving outcomes for patients with parasitic infections.

## DISCLOSURE

None.

## Data Availability

Data sharing is not applicable—no new data are generated.
